# JARID2 Is Involved in Transforming Growth Factor-Beta-Induced Epithelial-Mesenchymal Transition of Lung and Colon Cancer Cell Lines

**DOI:** 10.1371/journal.pone.0115684

**Published:** 2014-12-26

**Authors:** Shoichiro Tange, Dulamsuren Oktyabri, Minoru Terashima, Akihiko Ishimura, Takeshi Suzuki

**Affiliations:** Division of Functional Genomics, Cancer Research Institute, Kanazawa University, Kanazawa, Ishikawa, Japan; Seoul National University, Korea, Republic Of

## Abstract

Histone methylation plays a crucial role in various biological and pathological processes including cancer development. In this study, we discovered that JARID2, an interacting component of Polycomb repressive complex-2 (PRC2) that catalyzes methylation of lysine 27 of histone H3 (H3K27), was involved in Transforming Growth Factor-beta (TGF-ß)-induced epithelial-mesenchymal transition (EMT) of A549 lung cancer cell line and HT29 colon cancer cell line. The expression of *JARID2* was increased during TGF-ß-induced EMT of these cell lines and knockdown of *JARID2* inhibited TGF-ß-induced morphological conversion of the cells associated with EMT. *JARID2* knockdown itself had no effect in the expression of EMT-related genes but antagonized TGF-ß-dependent expression changes of EMT-related genes such as *CDH1*, *ZEB* family and *microRNA-200* family. Chromatin immunoprecipitation assays showed that JARID2 was implicated in TGF-ß-induced transcriptional repression of *CDH1* and *microRNA-200* family genes through the regulation of histone H3 methylation and EZH2 occupancies on their regulatory regions. Our study demonstrated a novel role of JARID2 protein, which may control PRC2 recruitment and histone methylation during TGF-ß-induced EMT of lung and colon cancer cell lines.

## Introduction

Lysine (K) methylation on the amino-terminal tail of histone H3 (K4, K9, K27 and K36) has emerged as an important post-translational modification due to its specific dynamics for transcriptional regulation [1], [2]. Methylation of H3K4 has been tightly linked to active transcription whereas methylation of H3K9 and H3K27 are repressive marks of chromatin. These modifications are regulated by histone lysine methyltransferases (KMTs) and lysine demethylases (KDMs). Recent studies have indicated that deregulation of these enzymes may contribute to the developmental defects and the pathogenesis of human diseases including cancer [1]–[3].

In order to find novel genes implicated in cancer development, we have performed retroviral insertional mutagenesis in mice. This screen led to the isolation of hundreds of candidate cancer genes including many genes encoding KMTs and KDMs [4], [5]. Previously, we reported that KDM5B/PLU1/JARID1B, an H3K4 demethylase and one of the candidate oncogenes, down-regulated the expression of *KAT5* and *CD82* genes to increase cell invasion [6] and repressed the expression of *microRNA-200* (*miR-200*) family, thereby promoting epithelial-mesenchymal transition (EMT) of cancer cells [7]. Thus our studies revealed that histone methyl-modifying enzymes were involved not only in tumor initiation but also in tumor progression.

Tumor progression has been associated with the activation of the EMT program that is induced by extrinsic signals such as Transforming Growth Factor-beta (TGF-ß) [8], [9]. EMT is characterized by the changes in epithelial and mesenchymal gene expression. Especially, the down-regulation of E-cadherin is essential for EMT. Several transcriptional repressors such as ZEB1 and ZEB2 are involved in E-cadherin transcriptional repression during EMT [10]. The reversible properties of EMT suggest that epigenetic regulation such as DNA methylation, histone modification and microRNA may be involved [11]. There are several papers including our study demonstrating the connection between E-cadherin repression and the function of histone methyl-modifying enzymes [7], [12], [13]. However, the role of histone methylation in EMT is just beginning to be uncovered.

JARID2 is one of the JARID proteins that contain JmjC (Jumonji C) domain and ARID (AT-rich interaction domain) [2]. However, JARID2 protein lacks histone demethylase activity characteristic of other JmjC domain proteins because it has amino acid substitutions in the conserved region [2]. JARID2 has been reported as an accessory component of Polycomb repressive complex-2 (PRC2) that regulates important gene expression patterns during development [14]. PRC2 contains the core subunits: EZH2, SUZ12, EED and RBBP4/7, and is responsible for repressive H3K27 methylation [15]. JARID2 was shown to control PRC2 occupancy and activity at the target genes in embryonic stem cells (ESCs) [16]–[19]. On the other hand, deregulation of PRC2 activity is thought to contribute to human cancer development. EZH2, an enzymatic component of PRC2, was shown to be overexpressed in metastatic cancer, and the expression levels were associated with tumor progression [20], [21]. However, the role of JARID2, an interacting component of PRC2, during cancer progression remains unknown.

In this study we investigated the function of JARID2 during TGF-ß-induced EMT of A549 lung cancer cell line and HT29 colon cancer cell line. We found that TGF-ß-dependent expression changes of EMT-related genes were inhibited by *JARID2* knockdown and enhanced by *JARID2* overexpression. Mechanistic investigations suggested that JARID2 was involved in TGF-ß-induced transcriptional repression of *CDH1* and *miR-200* family genes through the modulation of histone H3 methylation.

## Materials and Methods

### Plasmids

The small hairpin RNA (shRNA)-expressing retrovirus vectors were constructed as described previously [6]. The sense strand sequences of the oligonucleotides were as follows:

*JARID2* shRNA#1, 5′-ccgggaaacaggtttctaaggtaaactcgagtttaccttagaaacctgtttcttttt-3′

*JARID2* shRNA#2, 5′-ccgggcccaacagcatggtgtatttctcgagaaatacaccatgctgttgggcttttt-3′

The sequence of control shRNA was described previously [6]. We confirmed that the expression of *JARID2* was down-regulated with the infection of both *JARID2* shRNA-expressing retroviruses even in the presence of TGF-ß by quantitative RT-PCR (QRT-PCR) and Western blot (S1 Fig.). We also confirmed that both *JARID2* shRNAs caused the same effects in our EMT studies (S2 Fig.), and thus we presented the data of *JARID2* shRNA#1 as a representative result (described as JARID2 KD). Human *JARID2* cDNA was tagged with FLAG-6xHis-tag, and then cloned into pDON-5 Neo plasmid (Takara) to produce retroviruses expressing JARID2.

### Cell culture and transfection

A549 human lung cancer cell line and HT29 human colon cancer cell line were kindly provided by Dr. Y. Endo (Cancer Research Institute, Kanazawa Univ.) and maintained in Dulbecco's modified Eagle's medium (DMEM) with 10% FBS, 2 mM glutamine and penicillin/streptomycin (Sigma) at 37°C in 5% CO2. For EMT induction, A549 cells were treated with 1 ng/ml of TGF-ß (R&D Systems) for 12 to 72 hours, whereas HT29 cells were treated with 5 ng/ml of TGF-ß. The production and the infection procedures of shRNA or cDNA-expressing retroviruses were described previously [6].

### Quantitative PCR

RNA preparation and quantitative RT-PCR analysis were performed as described previously [6]. PCR data were normalized with respect to control human GAPDH expression. The averages from at least three independent experiments are shown with the standard deviations. *P*-values were calculated between control and the samples using Student's t-test. Primers used for the quantitative PCR were described previously [6], [7] and designed as follows:

Human *JARID2*, 5′- gagcatgtgtttcagcaagg-3′ and 5′- cttctcttccactagcctccag-3′

Human *JARID1A*, 5′- tgaacttctgtactgctgactgg-3′ and 5′- agccccacatctaagcattc-3′

Human *JARID1C*, 5′- ctacggaggaaccacagca-3′ and 5′- agcaggcagaagatgtggtag-3′

Human *JARID1D*, 5′- tcacagcagtgcccagttta-3′ and 5′- ggggtggtaacaagcagaag-3′

Human *Vimentin*, 5′- tacaggaagctgctggaagg-3′ and 5′ –accagagggagtgaatccag-3′

For microRNA quantification, TaqMan MicroRNA Assays (Applied Biosystems) for *miR-200a* (#000502) and *miR-200c* (#002300) were used. All data were normalized with respect to *RNU6B* (#001093) expression.

### Immunoblotting, cell staining and immuno-fluorescence assay

Immunoblotting was performed as described previously [7]. Anti-JARID2 (#NB100-2214, Novus Biologicals), anti-E-cadherin (#610181, BD Transduction Lab), anti-Fibronectin (SAB4500974, Sigma), anti-Vimentin (ab8069, Abcam), anti-ZEB1 (#3396, Cell Signaling), anti-ZEB2 (#61096, Active Motif), anti-phosphorylated SMAD3 (ab51451, Abcam) and anti-GAPDH (6C5, Millipore) antibodies were used. To detect the morphological changes of the cells, A549 or HT29 cells were stained with 0.4% crystal violet (Waldeck). To allow direct fluorescence of actin cytoskeleton, the cells were stained with 0.25 µM tetramethylrhodamine isothiocyanate (TRITC)-conjugated phalloidin (Sigma). For indirect immunofluorescence, the specimens were incubated with anti-E-cadherin antibody and treated with Alexa546-conjugated anti-mouse IgG antibody (Invitrogen). Nuclei were visualized with 4′,6-diamidino-2-phenylindole (DAPI).

### Chromatin immunoprecipitation (ChIP) assays

ChIP experiments were performed as previously described [6], [22]. The cross-linked chromatins were immunopreciptated with mouse antibody (anti-H3K27me3, anti-H3K4me3 [22], anti-EZH2 (#17-662, Millipore) and anti-FLAG (M2, #F1804, Sigma)). The enrichment of the specific amplified region was analyzed by quantitative PCR and percentage enrichment of each modification over input chromatin DNA was shown. Primers used for the quantitative PCR correspond to the region a of *CDH1* gene, region b of *miR-200b/a/429* gene, region b of *miR-200c/141* gene and region a of *GAPDH* gene, respectively, as described previously [7].

## Results

### The expression of JARID2 was increased during TGF-ß-induced EMT

**Figure 1:**
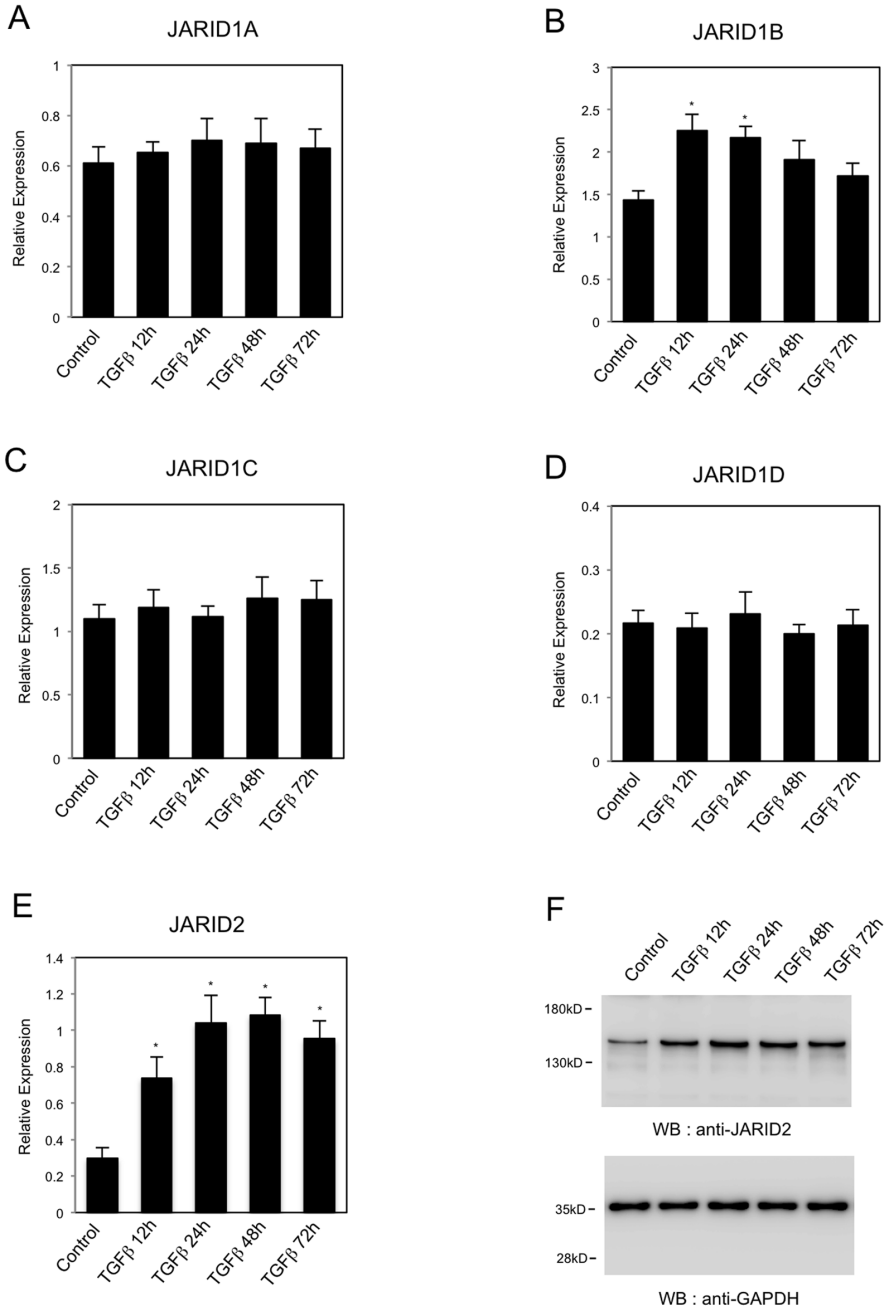
The expression of *JARID* genes during TGF-ß-induced EMT of A549 lung cancer cells. QRT-PCR analysis was performed to detect the expression of *JARID1A* (A), *JARID1B* (B), *JARID1C* (C), *JARID1D* (D) and *JARID2* (E) in A549 cells before and after the treatment of 1 ng/ml of TGF-ß (12 h, 24 h, 48 h and 72 h) (*, *P*<0.01 comparing to control). (F) Western blot was performed to detect the expression of JARID2 proteins during TGF-ß-induced EMT. As a control, anti-GAPDH antibody was used to show that equal amounts of proteins were loaded on the gel.

Previously, we have shown that KDM5B/PLU1/JARID1B played an important role in EMT of cancer cells [7]. JARID 1B is one of the JARID proteins that contain JmjC domain and ARID. To investigate the involvement of other JARID proteins in EMT, we first examined the changes in gene expression of *JARID* members during EMT process. We used a lung cancer cell line, A549, because it is a good EMT model system that shows clear morphological changes during EMT caused by TGF-ß treatment [23]. Quantitative RT-PCR (QRT-PCR) showed that *JARID1B* expression was significantly increased in A549 cells after the treatment of 1 ng/ml of TGF-ß (Fig. 1B), confirming the previous result [7]. For the expression of *JARID1A*, *JARID1C* and *JARID1D*, we did not observe any significant changes (Fig. 1A, 1C and 1D). However, *JARID2* expression was shown clearly increased by TGF-ß (Fig. 1E). We also examined the changes of protein expression for JARID2. Western blot showed that endogenous expression of JARID2 protein was significantly increased after TGF-ß treatment (Fig. 1F), suggesting its possible role in TGF-ß-induced EMT.

### Knockdown of JARID2 inhibited morphological changes of the cells induced by TGF-ß

**Figure 2:**
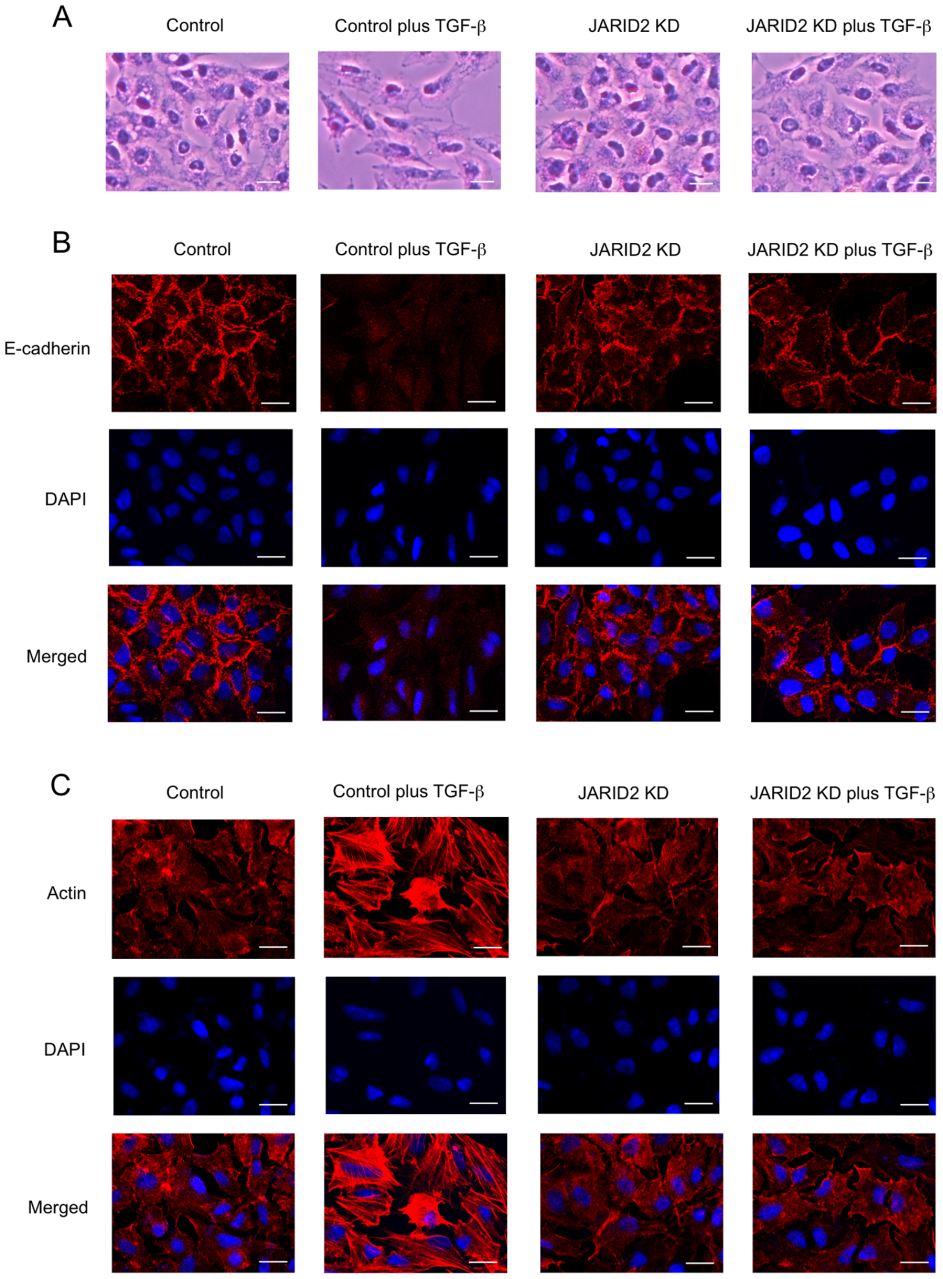
Knockdown of *JARID2* antagonized TGF-ß-induced morphological changes of A549 cells. (A) Cell morphological changes of A549 cells after TGF-ß treatment. A549 cells were infected with retroviruses expressing control shRNA or *JARID2* shRNA (described as *JARID2* KD) without or with the treatment of 1 ng/ml of TGF-ß for 48 hours. (B) Immunofluorescence images of cells showing the localization of E-cadherin. The panels of A549 cells with the same arrangement with (A) were stained with anti-E-cadherin antibody and with DAPI. (C) Fluorescence images of cells showing reorganization of actin cytoskeleton by staining with TRITC-phalloidin (Actin) and with DAPI. Scale bars: 20 µm.

To elucidate the function of JARID2 in EMT, we examined whether knockdown of *JARID2* would influence the EMT process induced by TGF-ß. A549 cells were infected with the control retrovirus or the retrovirus expressing *JARID2* shRNA, and the infected cells were treated with or without TGF-ß. After TGF-ß treatment, the control cells were dispersed, elongated and assumed a fibroblast-like appearance associated with EMT (Fig. 2A). *JARID2* knockdown itself did not change cell shapes significantly, but inhibited morphological changes of the cells induced by TGF-ß (Fig. 2A). Next we performed immunofluorescence assay using an antibody against E-cadherin, an epithelial cell marker. Untreated control A549 cells showed heterogeneous E-cadherin staining, and this staining was almost lost in TGF-ß-treated cells as described previously (Fig. 2B) [23]. As shown in Fig. 2B, E-cadherin staining was clearly detected in *JARID2* knockdown cells treated with or without TGF-ß, suggesting that the epithelial property might be maintained in *JARID2* knockdown cells even after TGF-ß treatment. We further examined the status of actin in the cells by TRITC-conjugated phalloidin staining, since actin reorganization occurs during EMT process [8]. In contrast to untreated control cells, TGF-ß dramatically induced actin fiber formation typical of EMT (Fig. 2C). However, in *JARID2* knockdown cells, we did not observe actin fiber formation even in the presence of TGF-ß (Fig. 2C).

**Figure 3:**
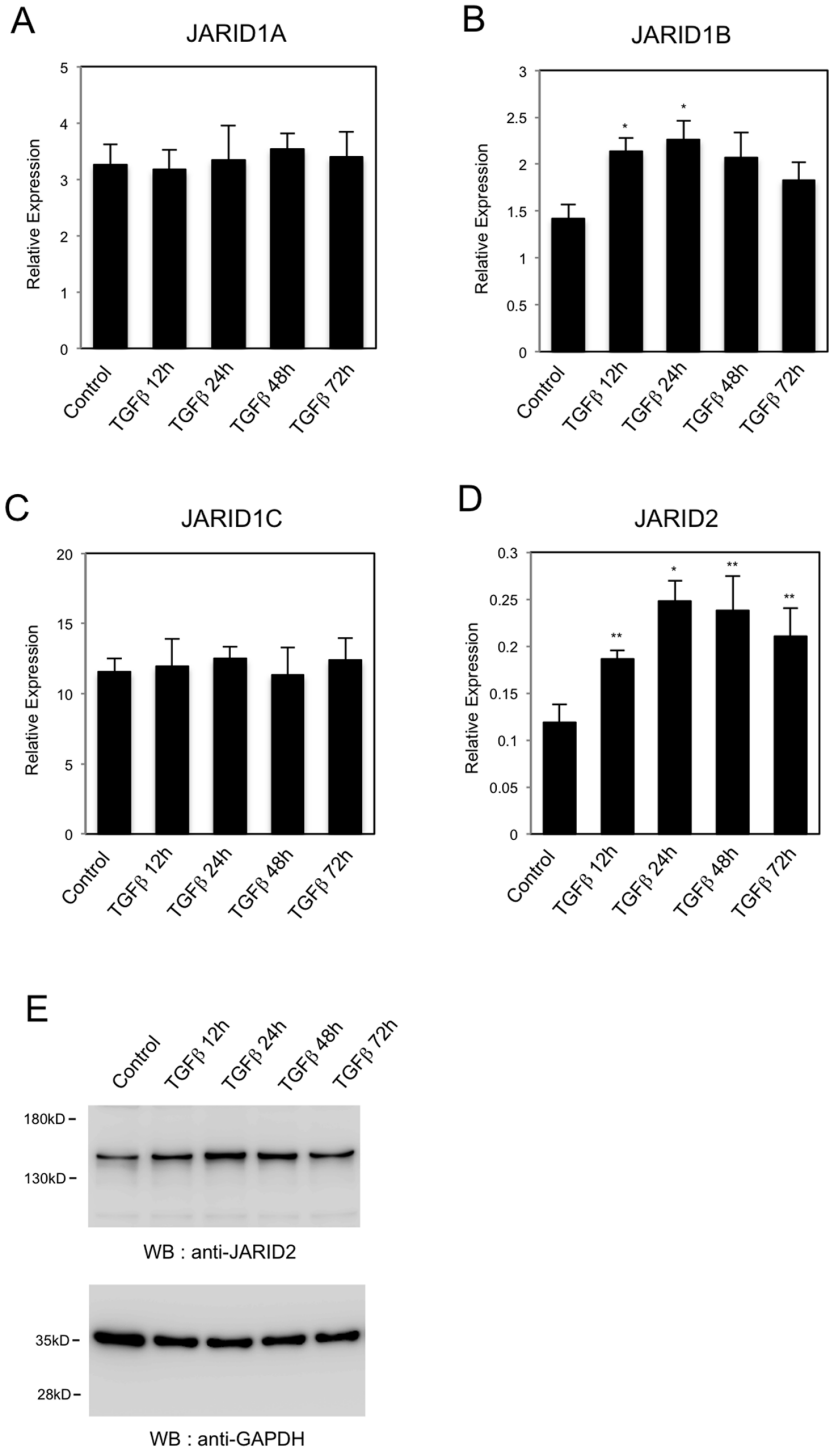
The expression of *JARID* genes during TGF-ß-induced EMT of HT29 colon cancer cells. QRT-PCR analysis was performed to detect the expression of *JARID1A* (A), *JARID1B* (B), *JARID1C* (C) and *JARID2* (D) in HT29 cells before and after the treatment of 5 ng/ml of TGF-ß (12 h, 24 h, 48 h and 72 h) (*, *P*<0.01 comparing to control; **, *P*<0.05 comparing to control). (E) Western blot was performed to detect the expression of JARID2 proteins during TGF-ß-induced EMT. As a control, anti-GAPDH antibody was used.

**Figure 4:**
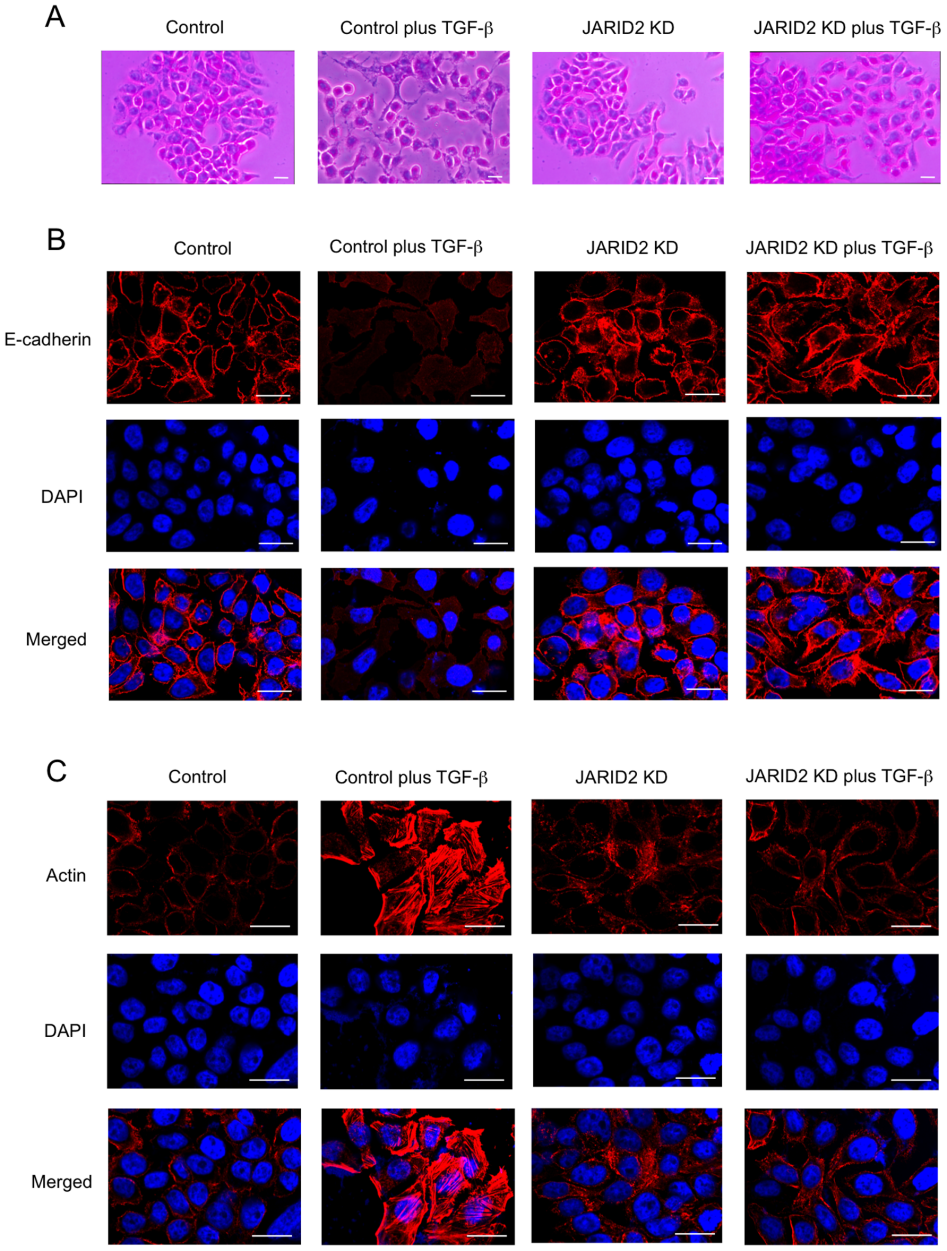
Knockdown of *JARID2* antagonized TGF-ß-induced morphological changes of HT29 cells. (A) Cell morphological changes of HT29 cells after TGF-ß treatment. HT29 cells were infected with retroviruses expressing control shRNA or *JARID2* shRNA (described as *JARID2* KD) without or with the treatment of 5 ng/ml of TGF-ß for 72 hours. (B) Immunofluorescence images of cells showing the localization of E-cadherin. The panels of HT29 cells with the same arrangement with (A) were stained with anti-E-cadherin antibody and with DAPI. (C) Fluorescence images of cells showing reorganization of actin cytoskeleton by staining with TRITC-phalloidin (Actin) and with DAPI. Scale bars: 20 µm.

We also examined whether *JARID2* knockdown would cause similar effects in another EMT model. We used a human colon cancer cell line, HT29, because it responds to TGF-ß for EMT [7]. For the expression of *JARID1A*, *JARID1B*, *JARID1C* and *JARID1D*, QRT-PCR showed that only *JARID1B* expression was significantly increased after the treatment of 5 ng/ml of TGF-ß (Fig. 3B). The expression of *JARID1D* was not detected in HT29 cells, because *JARID1D* gene is located on Y-chromosome and HT29 cells are derived from a female colon cancer patient. QRT-PCR and Western blot revealed that *JARID2* expression was also increased in HT29 cells after TGF-ß treatment (Fig. 3D and 3E). As shown in Fig. 4, TGF-ß treatment induced morphological changes, disappearance of E-cadherin staining and formation of actin stress fiber in HT29 cells. *JARID2* knockdown itself did not cause any significant changes compared to control cells, but antagonized these TGF-ß-induced phenotypes (Fig. 4). Altogether, these results indicated that knockdown of *JARID2* counteracted TGF-ß-induced morphological changes and cytoskeletal rearrangements of A549 and HT29 cancer cells characteristic of EMT.

### Knockdown of JARID2 affected the changes in expression of EMT-related genes induced by TGF-ß

**Figure 5:**
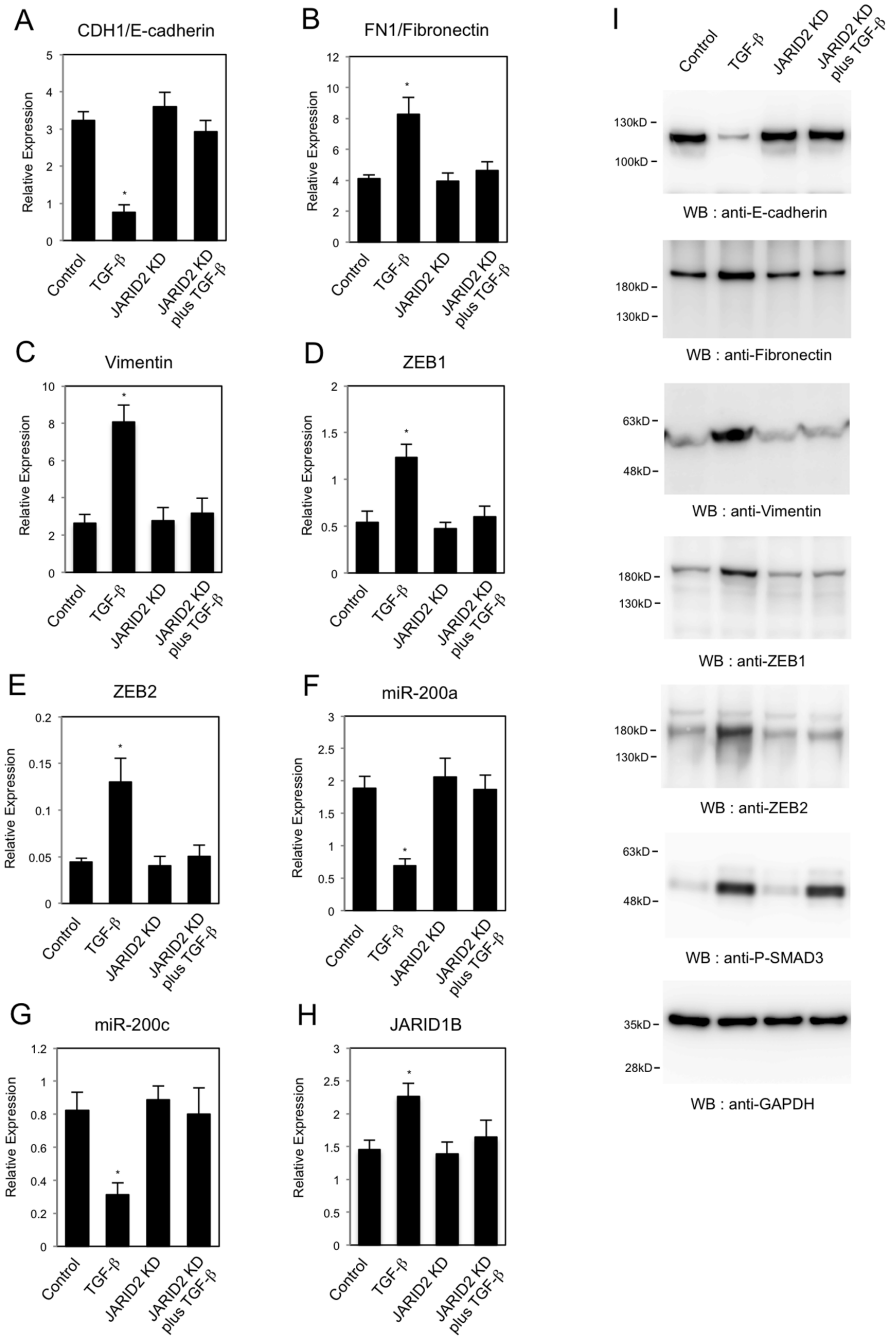
Knockdown of *JARID2* affected the TGF-ß-dependent changes in expression of EMT-related genes in A549 cells. QRT-PCR analysis was performed to detect the expression of *CDH1/E-cadherin* (A), *FN1/Fibronectin* (B), *Vimentin* (C), *ZEB1* (D), *ZEB2* (E), *miR-200a* (F), *miR-200c* (G) and *JARID1B* (H) in A549 cells infected with retroviruses expressing control shRNA or *JARID2* shRNA with or without the treatment of 1 ng/ml of TGF-ß for 24 hours (*, *P*<0.01 comparing to control). (I) Western blot was performed to detect the expression of E-cadherin, Fibronectin, Vimentin, ZEB1, ZEB2, phosphorylated SMAD3 (P-SMAD3) and GAPDH proteins using the corresponding antibodies.

EMT is characterized by the changes in epithelial and mesenchymal marker gene expression [8]. Thus we analyzed the expression of an epithelial marker, *CDH1/E-cadherin*, and mesenchymal markers, *FN1/Fibronectin* and *Vimentin* in the *JARID2* knockdown cells. QRT-PCR showed that TGF-ß decreased the expression of *CDH1/E-cadherin* mRNA in A549 cells (Fig. 5A) as previously reported [23]. *JARID2* knockdown itself had no effect on *CDH1* expression, but inhibited the repression of *CDH1* induced by TGF-ß (Fig. 5A). For *FN1/Fibronectin* and *Vimentin* whose expressions were up-regulated by TGF-ß, *JARID2* knockdown antagonized the effect of TGF-ß (Fig. 5B and 5C). These results suggested that *JARID2* knockdown inhibited the gene expression program of TGF-ß-induced EMT in A549 cells.

During EMT process, it has been reported that the expression of E-cadherin is regulated by the transcriptional repressors such as ZEB1 and ZEB2 [10]. Thus we analyzed the expression of ZEB family transcription repressors in the *JARID2* knockdown cells. As shown in Fig. 5D and 5E, TGF-ß treatment up-regulated the expression of *ZEB1* and *ZEB2* in A549 cells. *JARID2* knockdown itself did not affect the expression of *ZEB1* and *ZEB2* significantly, but inhibited the TGF-ß-dependent increase of both transcripts (Fig. 5D and 5E). This finding led us to investigate the possibility that the effect could be due to the regulation of *miR-200* family of microRNAs. The *miR-200* family has been reported to inhibit ZEB1 and ZEB2 specifically during EMT [24], [25]. Thus we examined whether *JARID2* knockdown would affect the expression of two representative miRNAs, *miR-200a* and *miR-200c*. Consistent with the previous reports [24], TGF-ß treatment resulted in the decreased expression of *miR-200a* and *miR-200c* in A549 cells (Fig. 5F and 5G). *JARID2* knockdown itself did not affect the expression, but inhibited the down-regulation of both microRNAs induced by TGF-ß (Fig. 5F and 5G). Next we examined the expression of *JARID1B* in the *JARID2* knockdown cells, because *JARID1B* was found to be up-regulated after TGF-ß treatment and to be implicated in EMT [7]. As shown in Fig. 5H, *JARID2* knockdown itself did not affect the expression of *JARID1B* significantly, but inhibited the TGF-ß-dependent increase of *JARID1B* transcript.

**Figure 6:**
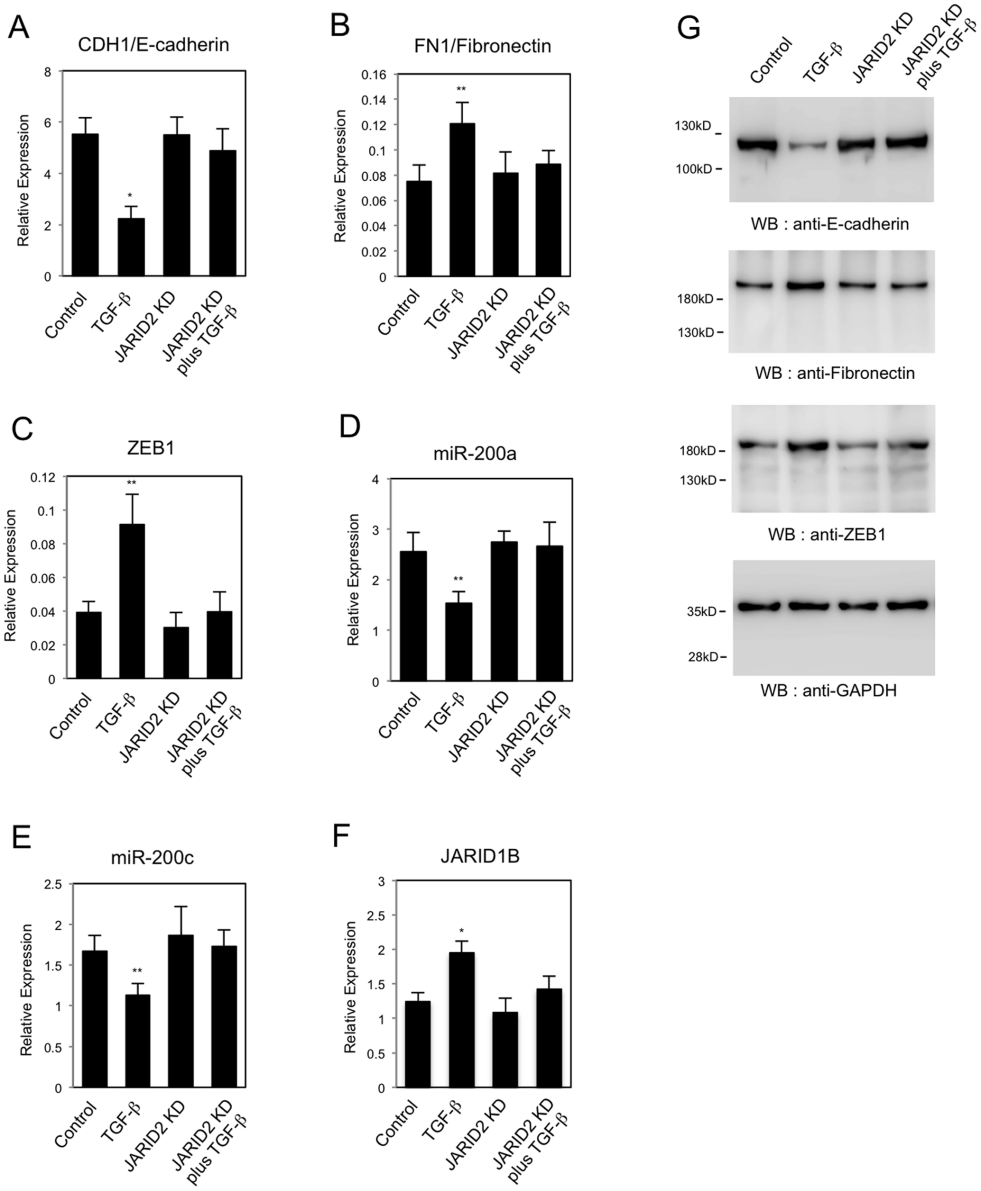
Knockdown of *JARID2* affected the TGF-ß-dependent changes in expression of EMT-related genes in HT29 cells. QRT-PCR analysis was performed to detect the expression of *CDH1/E-cadherin* (A), *FN1/Fibronectin* (B), *ZEB1* (C), *miR-200a* (D), *miR-200c* (E) and *JARID1B* (F) in HT29 cells infected with retroviruses expressing control shRNA or *JARID2* shRNA with or without the treatment of 5 ng/ml of TGF-ß for 24 hours (*, *P*<0.01 comparing to control; **, *P*<0.05 comparing to control). The expression of *Vimentin* and *ZEB2* was originally low or not detected in HT29 cells. (G) Western blot was performed to detect the expression of E-cadherin, Fibronectin, ZEB1 and GAPDH proteins using the corresponding antibodies.

We also analyzed the changes in protein expression for some of the EMT-related gene products in A549 cells. *JARID2* knockdown cancelled TGF-ß-dependent reduction of E-cadherin protein and increase of Fibronectin, Vimentin, ZEB1 and ZEB2 proteins (Fig. 5I), which enabled us to confirm the QRT-PCR results. Next we tried to examine whether TGF-ß signaling pathway would be impaired or not in the *JARID2* knockdown cells by detecting the phosphorylated SMAD3 proteins after TGF-ß treatment [9]. As shown in Fig. 5I, the phosphorylated SMAD3 proteins were induced by TGF-ß and their levels were similar in control cells and *JARID2* knockdown cells. This result indicated that activation of the downstream SMAD3 transcription factor by TGF-ß signal would not be impaired by *JARID2* knockdown. Moreover, we confirmed the effects of *JARID2* knockdown in the regulation of the EMT-related genes in another cancer cell line, HT29 (Fig. 6). QRT-PCR revealed that *JARID2* knockdown similarly inhibited TGF-ß-induced changes of *CDH1/E-cadherin*, *FN1/Fibronectin*, *ZEB1*, *miR-200a*, *miR-200c* and *JARID1B* expression in HT29 cells (Fig. 6A–F), and Western blot enabled us to confirm the QRT-PCR results (Fig. 6G). These results together suggested that JARID2 did not affect the activation of the downstream transcription factors by TGF-ß signal but was involved in TGF-ß-dependent transcriptional regulation of EMT-related genes in A549 lung cancer cell line and HT29 colon cancer cell line.

### JARID2 is implicated in the transcriptional regulation of CDH1 and miR-200 family gene by TGF-ß through the conversion of histone H3 methylation

JARID2 is an important cofactor of PRC2 enzyme complex that catalyzes methylation of H3K27 [14], [15] and involved in transcriptional repression in ESCs [16]–[19]. Thus we examined the methylated status of histone H3 on the regulatory regions of *CDH1* and *miR-200* family genes, which were transcriptionally repressed during TGF-ß-induced EMT, by chromatin immunoprecipitation (ChIP) assay. Genetically, the *miR-200* family is grouped in two polycistronic units: *miR-200b/200a/429* and *miR-200c/141* [26]. Following immunoprecipitation, the primer sets positioned upstream from the transcription start sites of *CDH1* gene and two microRNA clusters were used in quantitative PCR [7].

**Figure 7:**
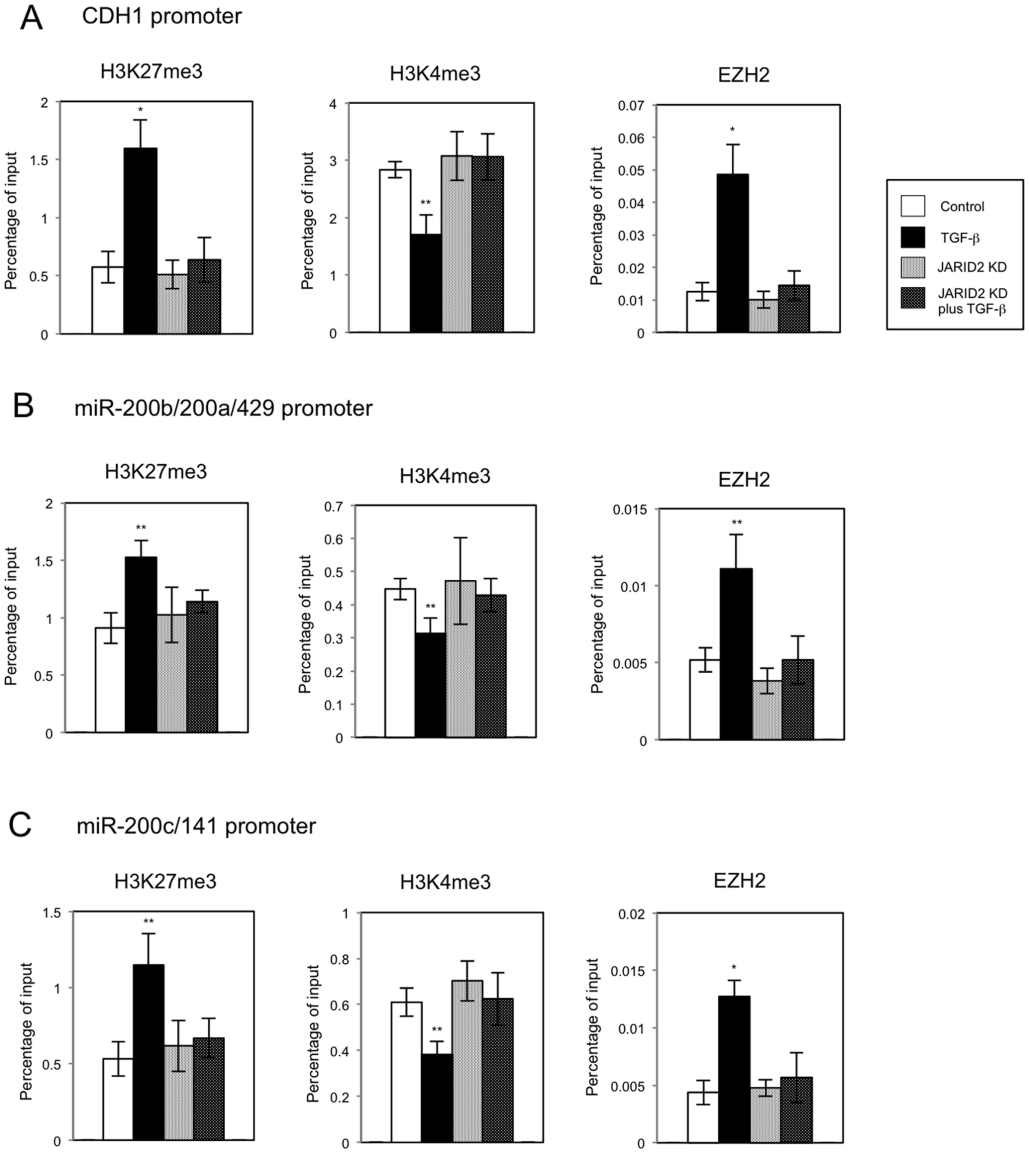
Knockdown of *JARID2* affected the TGF-ß-induced regulation of histone H3 methylation and EZH2 recruitment on the regulatory regions of *CDH1* gene and *miR-200* gene clusters in A549 cells. ChIP analyses of H3K27me3, H3K4me3 and EZH2 on the regulatory regions of *CDH1* (A), *miR-200b/200a/429* (B) and *miR-200c/141* genes (C) in A549 cells are shown. The occupancies of methylated histones or EZH2 protein on the regions were analyzed by quantitative PCR (*, *P*<0.01 comparing to control; **, *P*<0.05 comparing to control).

Because JARID2 can recruit PRC2 complex to the chromatin in ESCs [16]–[19], we first analyzed the transcriptionally repressive tri-methylated H3K27 (H3K27me3) status in A549 cells. On the regulatory regions of *CDH1*, *miR-200b/200a/429* and *miR-200c/141* genes, the levels of H3K27me3 were significantly increased after TGF-ß treatment (Fig. 7), which was correlated with the transcriptional repression of these genes. On the other hand, transcriptionally active H3K4me3 marks decreased substantially on these regulatory regions by TGF-ß (Fig. 7). More importantly, we observed the enhanced recruitment of EZH2, a catalytic subunit of PRC2 complex, on these regulatory regions after TGF-ß treatment (Fig. 7). These results suggested that TGF-ß-induced recruitment of EZH2 on the regulatory regions might be responsible for the transcriptional repression in A549 cells. *JARID2* knockdown itself did not cause any changes of histone methylation and EZH2 occupancies on these regions. Moreover, TGF-ß treatment in *JARID2* knockdown cells did not result in the increase of H3K27me3, the decrease of H3K4me3 and the increase of EZH2 recruitment (Fig. 7). This observation was correlated with the inhibition of TGF-ß-dependent transcriptional repression of *CDH1* and *miR-200* family genes in *JARID2* knockdown cells (Fig. 5A, 5F and 5G). We could not detect the recruitment of endogenous JARID2 protein on the regulatory regions by ChIP assay with the anti-JARID2 antibody, possibly due to its low reactivity for immunoprecipitation. However, these results suggested that JARID2 was responsible for TGF-ß-induced transcriptional repression of *CDH1* and *miR-200* family genes during EMT of A549 cells and that its function was associated with the regulation of recruitment of EZH2 on the chromatin for histone methylation. As a control experiment, we performed ChIP assay on the regulatory region of unrelated *GAPDH* gene in A549 cells. There were no significant changes in H3K27 and H3K4 methylation and EZH2 occupancies on the regulatory region of *GAPDH* gene by JARID2 knockdown and/or TGF-ß treatment (S3 Fig.). This indicated that JARID2-mediated changes of histone H3 methylation and EZH2 recruitment might be specific to the target genes regulated by JARID2 and TGF-ß, which was correlated with the specificity of transcriptional regulation.

**Figure 8:**
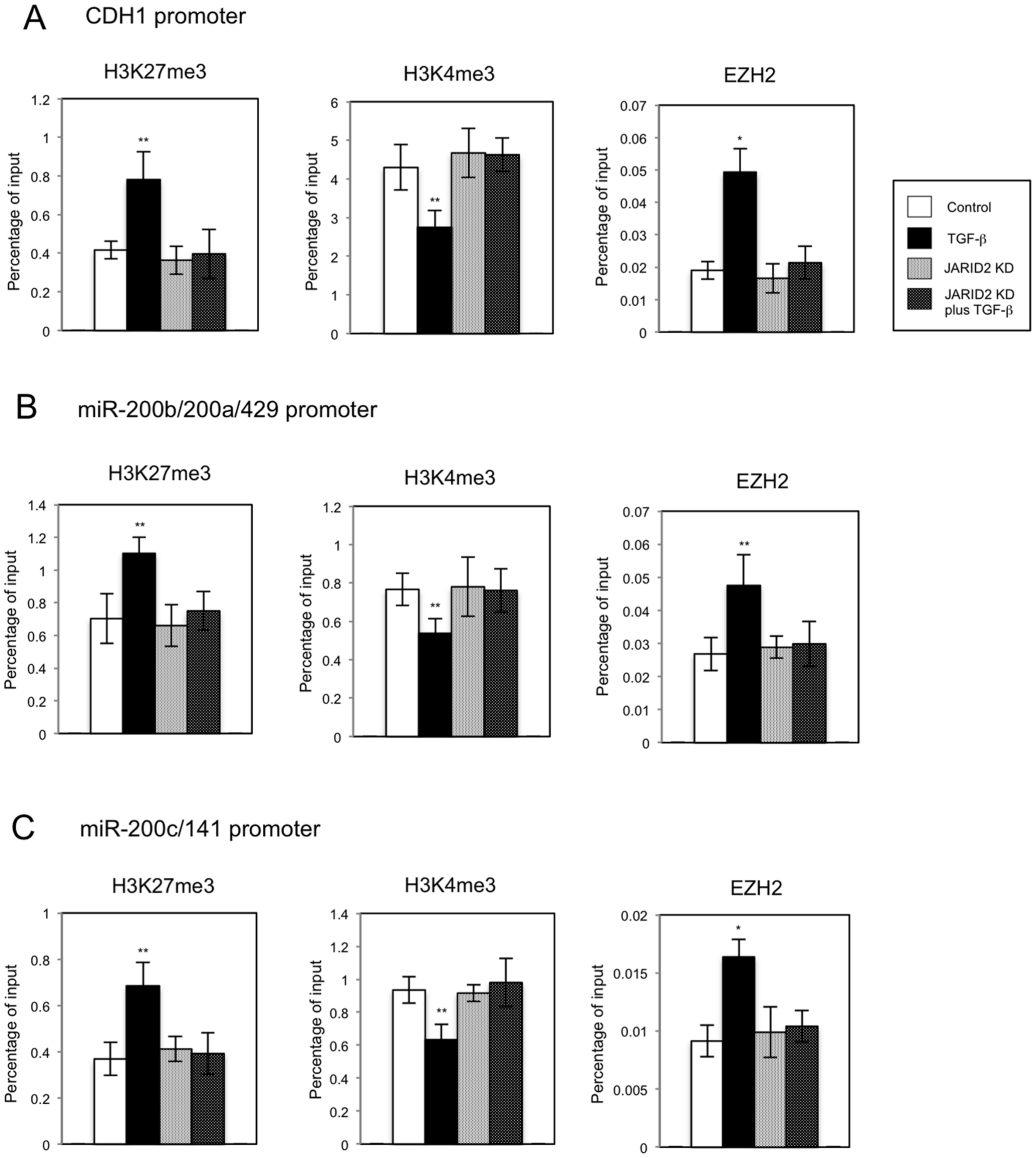
Knockdown of *JARID2* affected the TGF-ß-induced regulation of histone H3 methylation and EZH2 recruitment on the regulatory regions of *CDH1* gene and *miR-200* gene clusters in HT29 cells. ChIP analyses of H3K27me3, H3K4me3 and EZH2 on the regulatory regions of *CDH1* (A), *miR-200b/200a/429* (B) and *miR-200c/141* genes (C) in HT29 cells are shown. The occupancies of methylated histones or EZH2 protein on the regions were analyzed by quantitative PCR (*, *P*<0.01 comparing to control; **, *P*<0.05 comparing to control).

Moreover, we also examined the effects of *JARID2* knockdown in the status of histone H3 methylation and EZH2 recruitment on the regulatory regions of *CDH1* and *miR-200* family genes in another cancer cell line, HT29 (Fig. 8). ChIP analyses revealed that *JARID2* knockdown similarly inhibited TGF-ß-dependent increase of H3K27me3 and EZH2 occupancies and decrease of H3K4me3 on these regulatory regions (Fig. 8). These JARID2-mediated effects were not observed on the regulatory region of GAPDH gene (S4 Fig.). Therefore these results together suggested that JARID2 was required for TGF-ß-dependent changes of histone H3 methylation and EZH2 recruitment on the specific target genes during TGF-ß-induced EMT process of A549 and HT29 cancer cell lines.

### Over-expression of JARID2 enhanced TGF-ß-dependent transcriptional regulation of EMT-related genes

**Figure 9:**
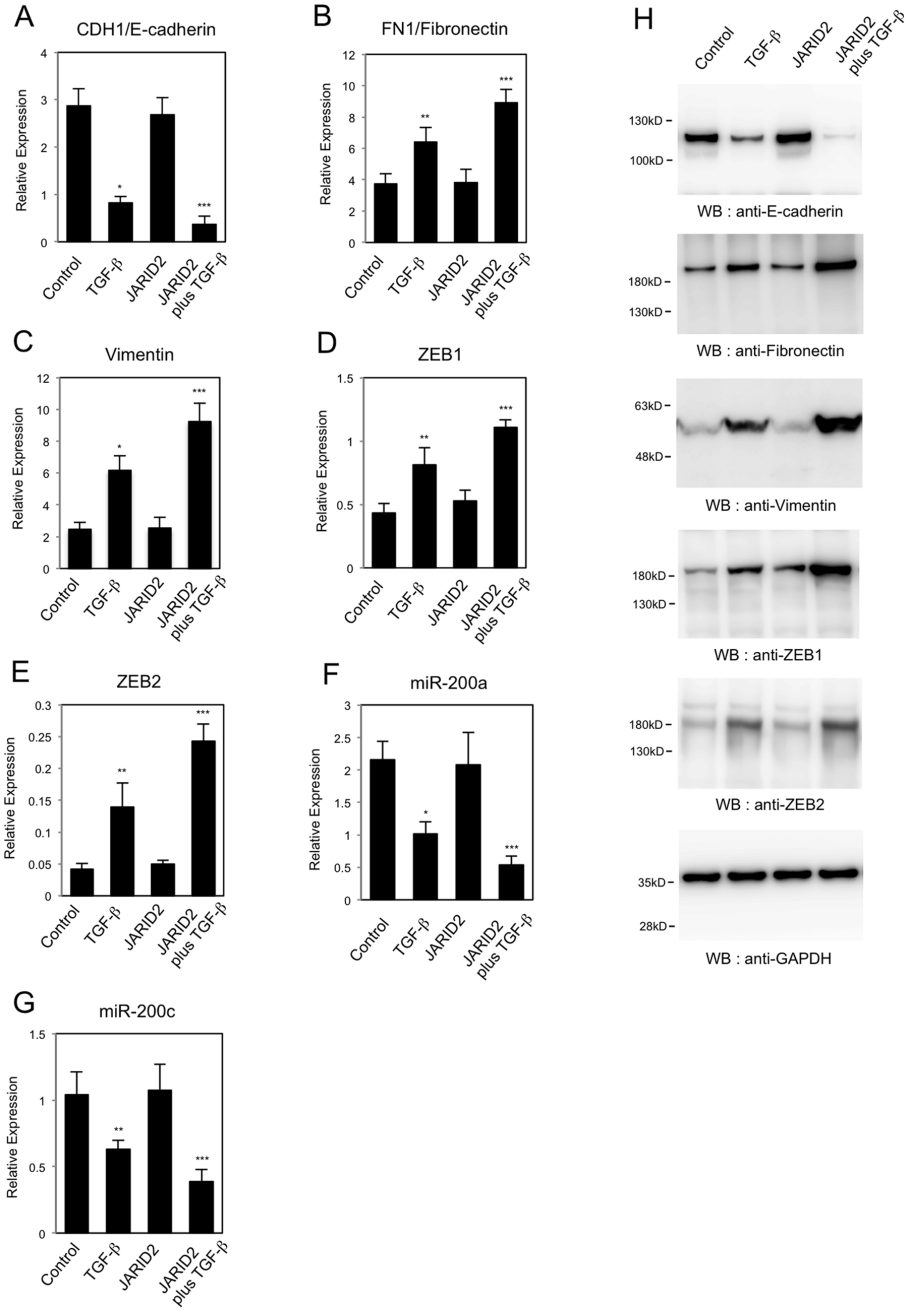
Over-expression of *JARID2* enhanced the TGF-ß-dependent changes in expression of EMT-related genes in A549 cells. QRT-PCR analysis was performed to detect the expression of *CDH1/E-cadherin* (A), *FN1/Fibronectin* (B), *Vimentin* (C), *ZEB1* (D), *ZEB2* (E), *miR-200a* (F) and *miR-200c* (G) in A549 cells infected with the control retrovirus or the retrovirus expressing *JARID2* with or without treatment of TGF-ß for 24 hours (*, *P*<0.01 comparing to control; **, *P*<0.05 comparing to control; ***, *P*<0.05 comparing to control plus TGF-ß). (H) Western blot was performed to detect the expression of E-cadherin, Fibronectin, Vimentin, ZEB1, ZEB2 and GAPDH proteins using the corresponding antibodies.

To extend our understanding for the regulation of EMT by JARID2, we examined the effects of *JARID2* over-expression in A549 cells. Over-expression of *JARID2* was confirmed by QRT-PCR and Western blot and the level of over-expressed *JARID2* was not significantly changed before and after TGF-ß treatment (S5 Fig.). Then we examined the expression of *CDH1/E-cadherin*, *FN1/Fibronectin*, *Vimentin*, *ZEB1, ZEB2, miR-200a* and *miR-200c* in A549 cells with *JARID2* over-expression. *JARID2* over-expression itself did not show any significant changes in the expression of EMT-related genes, but enhanced the effects of TGF-ß in the expression of EMT-related genes (Fig. 9A–G). In the *JARID2* over-expressing cells, the expression of *CDH1, miR-200a* and *miR-200c* was repressed more by TGF-ß, and the expression of *FN1*, *ZEB1* and *ZEB2* was activated more by TGF-ß (Fig. 9A–G). We also confirmed that *JARID2* over-expression enhanced the TGF-ß-dependent reduction of E-cadherin protein and increase of Fibronectin, Vimentin, ZEB1 and ZEB2 proteins (Fig. 9H). These results indicated that over-expression of *JARID2* potentiated TGF-ß-dependent transcriptional regulation during EMT process of A549 cells.

**Figure 10:**
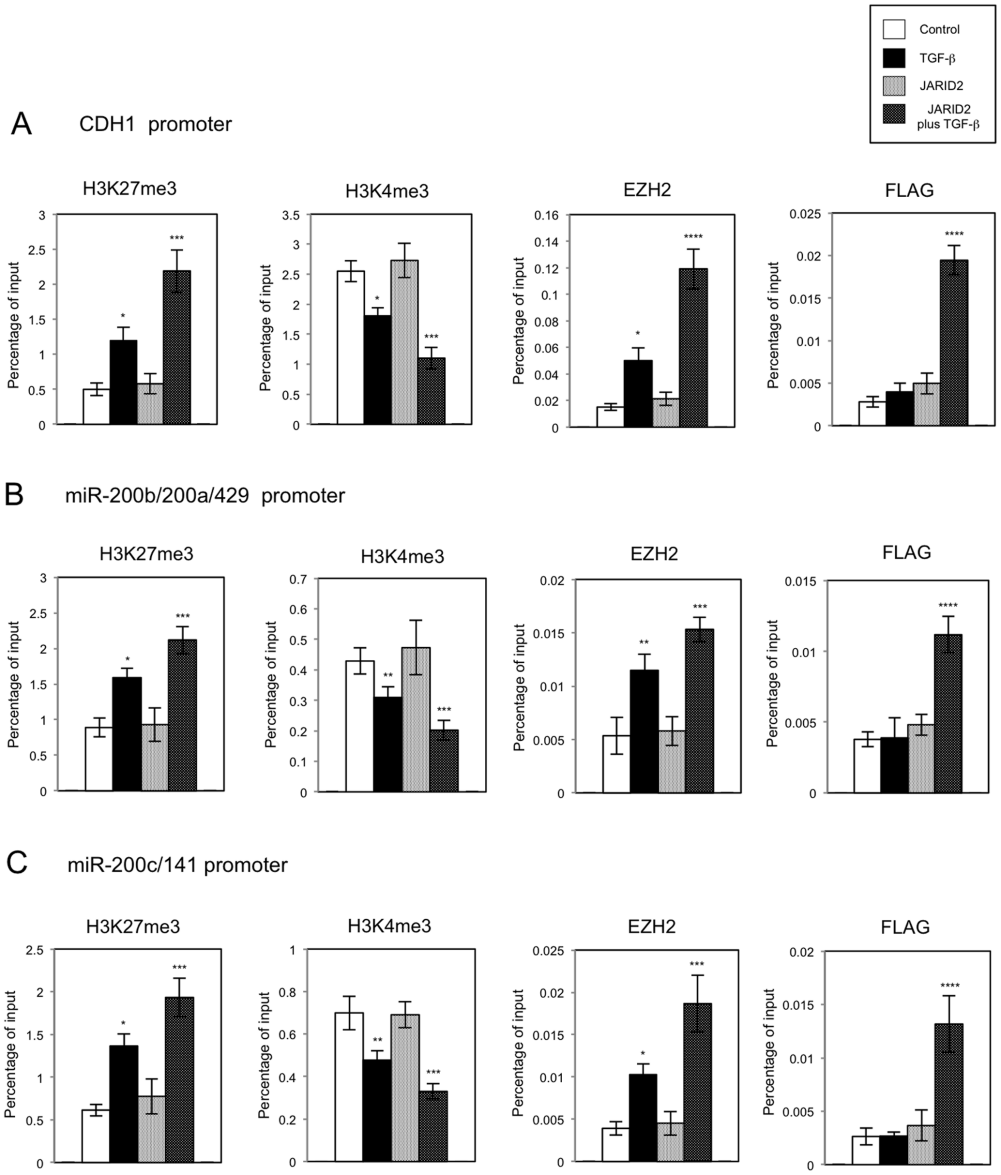
Over-expression of *JARID2* enhanced the TGF-ß-induced repression of *CDH1*, *miR-200a* and *miR-200c* through the regulation of histone H3 methylation in A549 cells. ChIP analyses of H3K27me3, H3K4me3, EZH2 and FLAG-tagged JARID2 on the regulatory regions of *CDH1* (A), *miR-200b/200a/429* (B) and *miR-200c/141* genes (C) in A549 cells are shown. The occupancies of methylated histones, EZH2 or FLAG-JARID2 protein on the regions were analyzed by quantitative PCR (*, *P*<0.01 comparing to control; **, *P*<0.05 comparing to control; ***, *P*<0.05 comparing to control plus TGF-ß; ****, *P*<0.01 comparing to control plus TGF-ß).

Next we tried to examine whether *JARID2* over-expression in A549 cells would affect the methylated status of histone H3 and the recruitment of EZH2 on the regulatory regions of *CDH1*, *miR-200b/200a/429* and *miR-200c/141* genes by ChIP analysis. On these regulatory regions, *JARID2* over-expression itself did not change the levels of H3K27me3 and H3K4me3 significantly, but enhanced the effects of TGF-ß on both modifications (Fig. 10), which was correlated well with the expression levels of *CDH1* and *miR-200* family genes. Furthermore, *JARID2* over-expression itself had little effect in EZH2 occupancies, but enhanced EZH2 recruitment on these regulatory regions after TGF-ß treatment (Fig. 10). We could also detect the increased recruitment of FLAG-tagged JARID2 proteins on the regions only in the presence of TGF-ß (Fig. 10). Again, we did not detect any significant changes in H3K27 and H3K4 methylation and EZH2 and FLAG-tagged JARID2 occupancies on the regulatory region of unrelated *GAPDH* gene (S6 Fig.). Altogether, these results suggested that JARID2 was directly involved in the recruitment of EZH2 on the regulatory regions of *CDH1*, *miR-200b/200a/429* and *miR-200c/141* genes for transcriptional repression in A549 cells, which was highly dependent on TGF-ß signal.

## Discussion

In this study, we found that knockdown of *JARID2* antagonized TGF-ß-induced EMT of A549 lung cancer cell line and HT29 colon cancer cell line by inhibiting TGF-ß-dependent changes in expression of EMT-related genes such as *CDH1*, *ZEB* family and *miR-200* family. On the other hand, overexpression of *JARID2* was shown to enhance the TGF-ß-induced expression changes of EMT-related genes. ChIP analyses revealed that JARID2 might induce the increase of EZH2 recruitment and histone H3K27 methylation on the regulatory regions of *CDH1* and *miR-200* family genes in the presence of TGF-ß, thereby causing TGF-ß-dependent transcriptional repression. Our study uncovers a novel role of JARID2, an accessary component of PRC2 complex, in TGF-ß-dependent EMT of lung and colon cancer cell lines, and has important implication in targeting cancer progression.

Methylation of histone H3K27 is an important modification implicated in development, stemness and cancer [15]. The EZH2-containing PRC2 complex regulates H3K27 methylation for gene silencing. Over-expression of *EZH2* was involved in tumor initiation and progression [21]. It was reported that EZH2 could repress the expression of *CDH1/E-cadherin* and *miR-200* family genes possibly through the regulation of H3K27 methylation [12], [27]. Moreover, a genome-wide profiling of histone methylation during EMT revealed strong correlations between histone methylations and gene expression [28]. For certain target genes that have both modifications of H3K27me3 and H3K4me3, the transcription level was dependent on the relative intensities of repressive H3K27me3 and active H3K4me3 marks. These previous findings were consistent with our ChIP results: TGF-ß-induced the increase of EZH2 occupancies and H3K27me3 and the decrease of H3K4me3 on the *CDH1*, *miR-200b/200a/429* and *miR-200c/141* genes, resulting in transcriptional repression, but *JARID2* knockdown antagonized TGF-ß-induced epigenetic changes, consequently inhibiting transcriptional repression. Therefore, this study validated the importance of H3K27 methylation during EMT program and demonstrated novel regulation for H3K27 methylation and PRC2 function mediated by JARID2.

In this study, we focused on ZEB family transcription factors in the regulation of *CDH1/E-cadherin* expression and elucidated the underlying mechanism through epigenetic regulation of *miR-200* family during TGF-ß-induced EMT of A549 and HT29 cells. However, we could not rule out the possibilities that other pathways might also contribute to TGF-ß-induced EMT process. There are other transcription factors such as SNAIL1, SNAIL2 and TWIST that are implicated in transcriptional repression of *CDH1/E-cadherin* during EMT process. Our preliminary experiments showed that *JARID2* knockdown could also antagonize the effect of TGF-ß in the induction of *SNAIL1* and *SNAIL2*, and that *TWIST* expression was extremely low and not responded to TGF-ß in A549 and HT29 cells. Currently we have not identified the precise mechanism by which *JARID2* knockdown inhibits TGF-ß-mediated induction of *SNAIL* family. Since the *SNAIL1/miR-34* pathway is another important feedback loop regulating EMT [29], epigenetic regulation of *miR-34* would be one of the promising targets. Further experiments will be necessary to clarify the whole molecular events that are critical in TGF-ß-induced EMT.

We also found that *JARID1B* H3K4 demethylase was significantly up-regulated after TGF-ß treatment and this increase was inhibited by *JARID2* knockdown in A549 and HT29 cells. Previously, we showed that *JARID1B* was not required for TGF-ß-induced EMT based on the result that *JARID1B* knockdown could not block TGF-ß-induced EMT of A549 cells [7]. Therefore, it is unlikely that the inhibition of *JARID1B* induction is primarily responsible for the inhibition of TGF-ß-induced EMT by *JARID2* knockdown. However, our experiments indicated that methylation of H3K4 on the specific target genes was dynamically changed during TGF-ß-induced EMT of A549 and HT29 cells (Figs. 7 and 8) and *JARID1B* over-expression promoted EMT process of both cells [7], which suggested an important role of H3K4-methyl-modifying enzymes during this process. Thus we will need more studies for the functional interaction of JARID2-containing PRC2 complex and H3K4-methyl-modifying enzymes to clarify the epigenetic regulation of TGF-ß-induced EMT in detail.

Increasing evidence indicates that deregulation of enzymes and cofactors engaged in histone methylation has been associated with the initiation and progression of many human cancers [1]–[3]. However, their roles in cancer development are sometimes controversial. *EZH2* over-expression has been found in a number of tumors and correlated with poor prognosis [20], [21]. However, large-scale cancer genome sequencing identified coding mutations within *EZH2* gene in various myeloid and lymphoid neoplasms, suggesting a tumor-suppressive role for EZH2 [30]. In the case of JARID2, a previous report showed a strong association of *JARID2* gene deletion with leukemic transformation of chronic myeloid malignancies, confirming tumor-suppressive function of PRC2 members in these cell lineages [31]. In contrast, our study proved a novel function of JARID2 in TGF-ß-induced EMT of lung and colon cancer cell lines. Furthermore, a recent paper reported that higher levels of *JARID2* expression were associated with metastasis at diagnosis in rhabdomyosarcomas, supporting the role of JARID2 in tumor progression [32]. These results suggest that the roles of these epigenetic regulators in tumor development are significantly dependent on many factors and cellular context. Therefore, careful analyses on different biological effects should be performed to understand the underlying mechanism of action.

Previous studies regarding JARID2 function in ESCs revealed that *JARID2* knockdown resulted in a marked decrease of PRC2 components on the target genes in ESCs [16]–[19]. This result seems contrary to our finding that *JARID2* knockdown itself did not cause any significant changes of histone methylation and EZH2 occupancies on the regulatory regions of *CDH1* and *miR-200* genes in A549 lung cancer cells and HT29 colon cancer cells. Based on our results, the observed function of JARID2 during EMT was highly dependent on TGF-ß signal. This discrepancy suggests that JARID2-mediated PRC2 recruitment might involve distinct mechanisms in ESCs and more differentiated cells. It has been proposed that cells undergoing EMT acquire stem cell properties [33]. TGF-ß treatment in A549 and HT29 cells might create stem cell-like environment during EMT, in which specific functional interaction of JARID2 and PRC2 complex would be effective. Furthermore, several reports regarding the regulation of JARID2 and PRC2 by long noncoding RNAs (lncRNAs) might provide another important clue [34]–[36]. They demonstrated that JARID2 binding to lncRNA was necessary for proper recruitment of PRC2 on the target genes in ESCs. In this study, we observed that *JARID2* over-expression itself had little effect in the levels of H3 methylation and EZH2 occupancies on the regulatory regions, but enhanced the effects of TGF-ß. These results suggested that some additional factors and/or signals induced by TGF-ß might be required for JARID2 to regulate the recruitment and activation of PRC2 complex on the target genes. Currently, the activation mechanisms of JARID2 and PRC2 during TGF-ß-induced EMT remain elusive. Further studies will be required to extend our understanding.

We have discovered a novel role of JARID2 in TGF-ß-induced EMT of A549 lung cancer cell line and HT29 colon cancer cell line. JARID2 was involved in EMT by regulating TGF-ß-dependent changes in expression of EMT-related genes through the modulation of PRC2 recruitment and histone methylation. Our findings strongly suggest that alteration of epigenetic regulation such as histone methylation and microRNA expression contributes to a critical step for malignant progression of cancer. Thus this study can propose the attractive targets for therapeutic interventions during malignant tumor progression.

## Supporting Information

S1 Fig **The expression of endogenous** ***JARID2*** **in A549 cells is efficiently down-regulated by its shRNAs.** (A) A549 cells were infected with the control retrovirus or the retrovirus expressing each *JARID2* shRNA (*JARID2* sh1 and sh2). The infected cells were treated with or without TGF-ß for 48 hours. The expression of *JARID2* mRNAs was detected by QRT-PCR (*, *P*<0.01 comparing to control). (B) Western blotting was performed to detect the expression of JARID2 proteins. As a control, anti-GAPDH antibody was used to show that equal amounts of proteins were loaded on the gel.
(DOCX)

S2 Fig **Both shRNAs for** ***JARID2*** **caused essentially the same effects in the expression of EMT-related genes induced by TGF-ß.** QRT-PCR analysis was performed to detect the expression of *CDH1/E-cadherin* (A), *FN1/Fibronectin* (B), *ZEB1* (C) and *ZEB2* (D) in A549 cells infected with retroviruses expressing control shRNA, *JARID2* shRNA#1 or *JARID2* shRNA#2 with or without treatment of TGF-ß (*, *P*<0.01 comparing to control).
(DOCX)

S3 Fig **Knockdown of** ***JARID2*** **did not affect the histone H3 methylation and EZH2 recruitment on the regulatory region of** ***GAPDH*** **gene in A549 cells.** ChIP analyses of H3K27me3, H3K4me3 and EZH2 on the regulatory region of *GAPDH* gene in A549 cells are shown. The occupancies of methylated histones or EZH2 protein on the region were analyzed by quantitative PCR.
(DOCX)

S4 Fig **Knockdown of** ***JARID2*** **did not affect the histone H3 methylation and EZH2 recruitment on the regulatory region of** ***GAPDH*** **gene in HT29 cells.** ChIP analyses of H3K27me3, H3K4me3 and EZH2 on the regulatory region of *GAPDH* gene in HT29 cells are shown. The occupancies of methylated histones or EZH2 protein on the region were analyzed by quantitative PCR.
(DOCX)

S5 Fig **Over-expression of** ***JARID2*** **was detected in A549 cells.** QRT-PCR analysis (A) and Western blot (B) were performed to detect the expression of *JARID2* in A549 cells infected with the control retrovirus or the retrovirus expressing *JARID2* with or without the treatment of 1 ng/ml of TGF-ß for 24 hours (*, *P*<0.01 comparing to control).
(DOCX)

S6 Fig **Over-expression of** ***JARID2*** **did not affect the histone H3 methylation and EZH2 recruitment on the regulatory region of** ***GAPDH*** **gene in A549 cells.** ChIP analyses of H3K27me3, H3K4me3, EZH2 and FLAG-tagged JARID2 on the regulatory region of *GAPDH* gene in A549 cells are shown. The occupancies of methylated histones, EZH2 or FLAG-JARID2 protein on the region were analyzed by quantitative PCR.
(DOCX)
