# Supplementary material for: JARID2 Is Involved in Transforming Growth Factor-Beta-Induced Epithelial-Mesenchymal Transition of Lung and Colon Cancer Cell Lines
Source: PLoS One. 2014 Dec 26;9(12):e115684. doi: 10.1371/journal.pone.0115684 (PMC4277293; doi:10.1371/journal.pone.0115684)
Supplement: S1 Fig — The expression of endogenous JARID2 in A549 cells is efficiently down-regulated by its shRNAs. (A) A549 cells were infected with the control retrovirus or the retrovirus expressing each JARID2 shRNA (JARID2 sh1 and sh2). The infected cells were treated with or without TGF-ß for 48 hours. The expression of JARID2 mRNAs was detected by QRT-PCR (*, P<0.01 comparing to control). (B) Western blotting was performed to detect the expression of JARID2 proteins. As a control, anti-GAPDH antibody was used to show that equal amounts of proteins were loaded on the gel. (DOCX) [file pone.0115684.s001.docx]

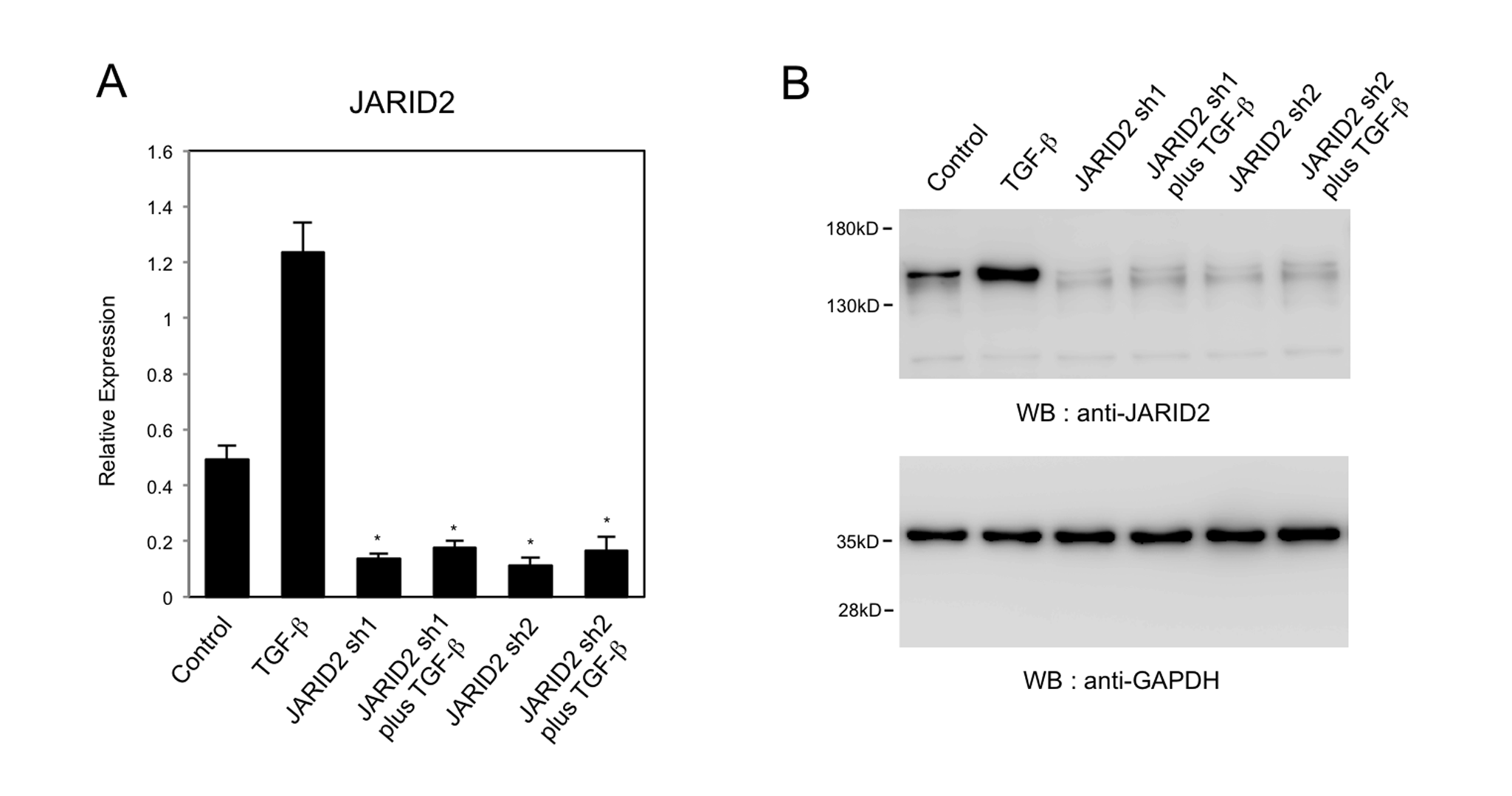


Figure S1. The expression of endogenous *JARID2* in A549 cells is efficiently down-regulated by its shRNAs.

(A) A549 cells were infected with the control retrovirus or the retrovirus expressing each *JARID2* shRNA (*JARID2* sh1 and sh2). The infected cells were treated with or without TGF-β for 48 hours. The expression of *JARID2* mRNAs was detected by QRT-PCR (*, *P* < 0.01 comparing to control). (B) Western blotting was performed to detect the expression of JARID2 proteins. As a control, anti-GAPDH antibody was used to show that equal amounts of proteins were loaded on the gel.
