# Supplementary material for: JARID2 Is Involved in Transforming Growth Factor-Beta-Induced Epithelial-Mesenchymal Transition of Lung and Colon Cancer Cell Lines
Source: PLoS One. 2014 Dec 26;9(12):e115684. doi: 10.1371/journal.pone.0115684 (PMC4277293; doi:10.1371/journal.pone.0115684)
Supplement: S2 Fig — Both shRNAs for JARID2 caused essentially the same effects in the expression of EMT-related genes induced by TGF-ß. QRT-PCR analysis was performed to detect the expression of CDH1/E-cadherin (A), FN1/Fibronectin (B), ZEB1 (C) and ZEB2 (D) in A549 cells infected with retroviruses expressing control shRNA, JARID2 shRNA#1 or JARID2 shRNA#2 with or without treatment of TGF-ß (*, P<0.01 comparing to control). (DOCX) [file pone.0115684.s002.docx]

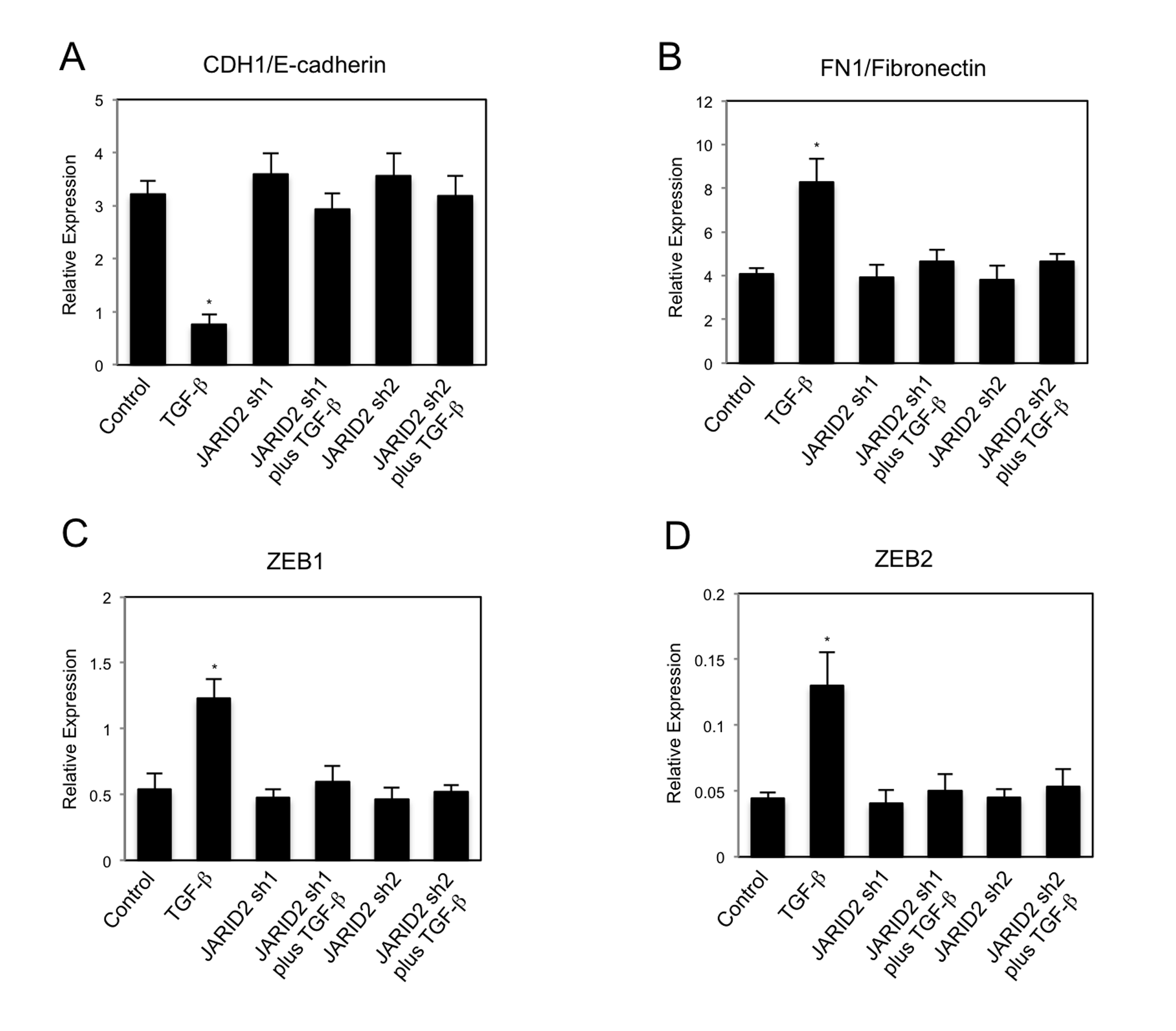


Figure S2. Both shRNAs for *JARID2* caused essentially the same effects in the expression of EMT-related genes induced by TGF-β.

QRT-PCR analysis was performed to detect the expression of *CDH1/E-cadherin* (A), *FN1/Fibronectin* (B), *ZEB1* (C) and *ZEB2* (D) in A549 cells infected with retroviruses expressing control shRNA, *JARID2* shRNA#1 or *JARID2* shRNA#2 with or without treatment of TGF-β (*, *P* < 0.01 comparing to control).
