# Supplementary material for: JARID2 Is Involved in Transforming Growth Factor-Beta-Induced Epithelial-Mesenchymal Transition of Lung and Colon Cancer Cell Lines
Source: PLoS One. 2014 Dec 26;9(12):e115684. doi: 10.1371/journal.pone.0115684 (PMC4277293; doi:10.1371/journal.pone.0115684)
Supplement: S4 Fig — Knockdown of JARID2 did not affect the histone H3 methylation and EZH2 recruitment on the regulatory region of GAPDH gene in HT29 cells. ChIP analyses of H3K27me3, H3K4me3 and EZH2 on the regulatory region of GAPDH gene in HT29 cells are shown. The occupancies of methylated histones or EZH2 protein on the region were analyzed by quantitative PCR. (DOCX) [file pone.0115684.s004.docx]

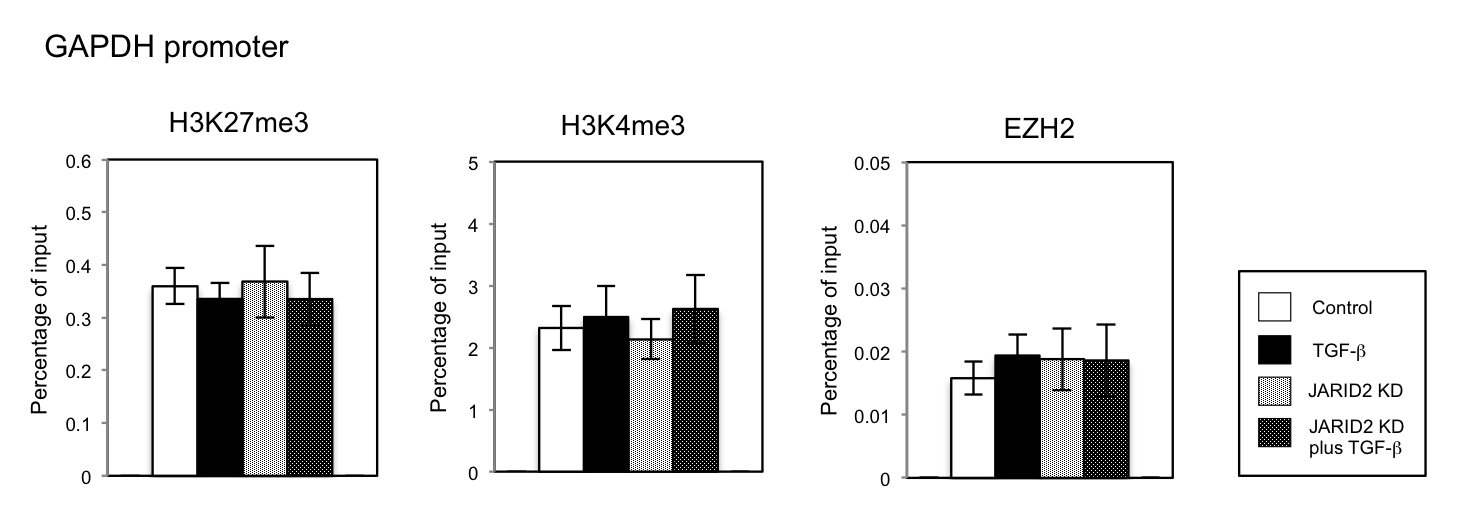


Figure S4. Knockdown of *JARID2* did not affect the histone H3 methylation and EZH2 recruitment on the regulatory region of *GAPDH* gene in HT29 cells.

ChIP analyses of H3K27me3, H3K4me3 and EZH2 on the regulatory region of *GAPDH* gene in HT29 cells are shown. The occupancies of methylated histones or EZH2 protein on the region were analyzed by quantitative PCR.
