# Supplementary material for: JARID2 Is Involved in Transforming Growth Factor-Beta-Induced Epithelial-Mesenchymal Transition of Lung and Colon Cancer Cell Lines
Source: PLoS One. 2014 Dec 26;9(12):e115684. doi: 10.1371/journal.pone.0115684 (PMC4277293; doi:10.1371/journal.pone.0115684)
Supplement: S5 Fig — Over-expression of JARID2 was detected in A549 cells. QRT-PCR analysis (A) and Western blot (B) were performed to detect the expression of JARID2 in A549 cells infected with the control retrovirus or the retrovirus expressing JARID2 with or without the treatment of 1 ng/ml of TGF-ß for 24 hours (*, P<0.01 comparing to control). (DOCX) [file pone.0115684.s005.docx]

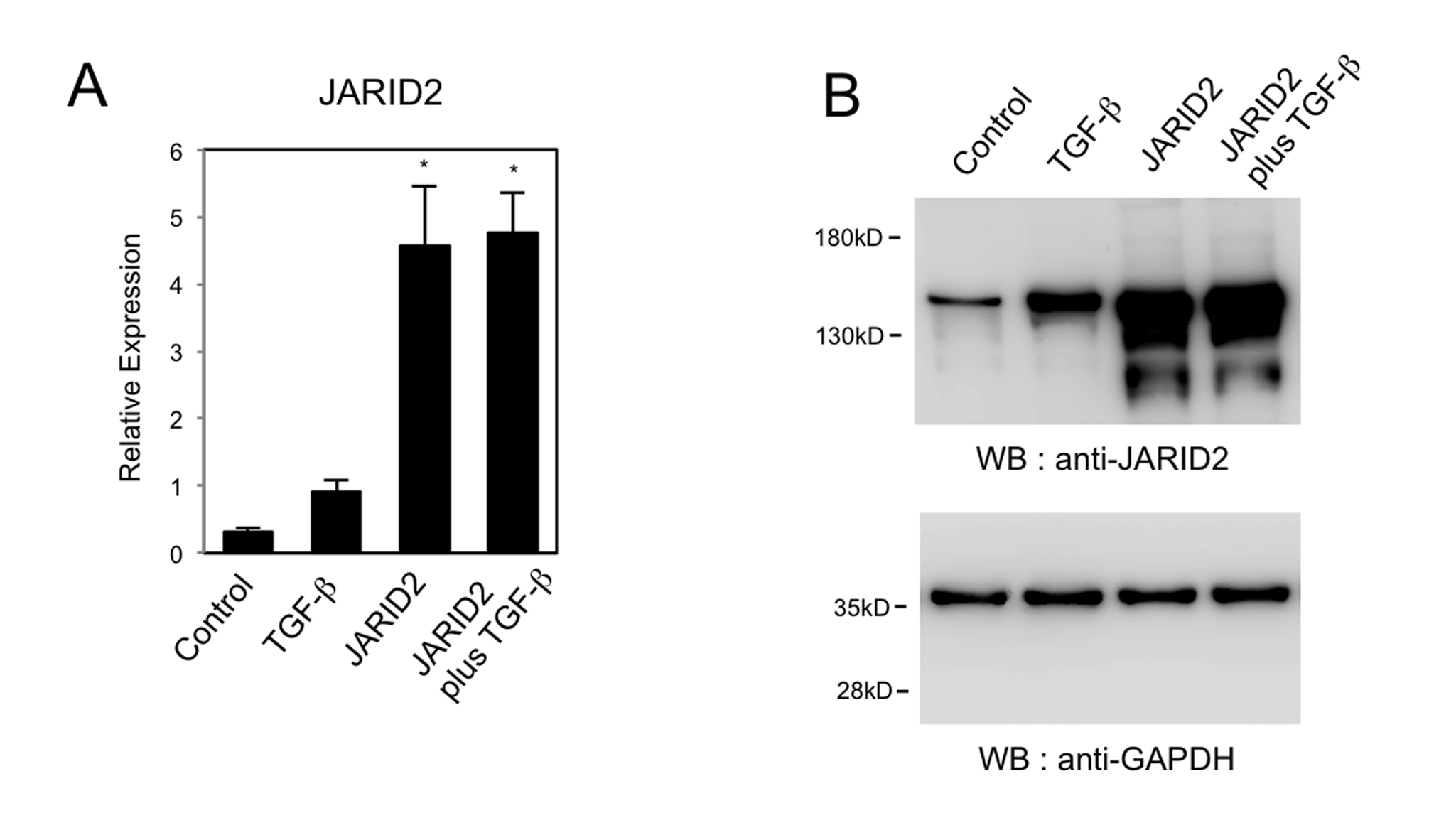


Figure S5. Over-expression of *JARID2* was detected in A549 cells.

QRT-PCR analysis (A) and Western blot (B) were performed to detect the expression of *JARID2* in A549 cells infected with the control retrovirus or the retrovirus expressing *JARID2* with or without the treatment of 1 ng/ml of TGF-β for 24 hours (*, *P* < 0.01 comparing to control).
