# Supplementary material for: JARID2 Is Involved in Transforming Growth Factor-Beta-Induced Epithelial-Mesenchymal Transition of Lung and Colon Cancer Cell Lines
Source: PLoS One. 2014 Dec 26;9(12):e115684. doi: 10.1371/journal.pone.0115684 (PMC4277293; doi:10.1371/journal.pone.0115684)
Supplement: S6 Fig — Over-expression of JARID2 did not affect the histone H3 methylation and EZH2 recruitment on the regulatory region of GAPDH gene in A549 cells. ChIP analyses of H3K27me3, H3K4me3, EZH2 and FLAG-tagged JARID2 on the regulatory region of GAPDH gene in A549 cells are shown. The occupancies of methylated histones, EZH2 or FLAG-JARID2 protein on the region were analyzed by quantitative PCR. (DOCX) [file pone.0115684.s006.docx]

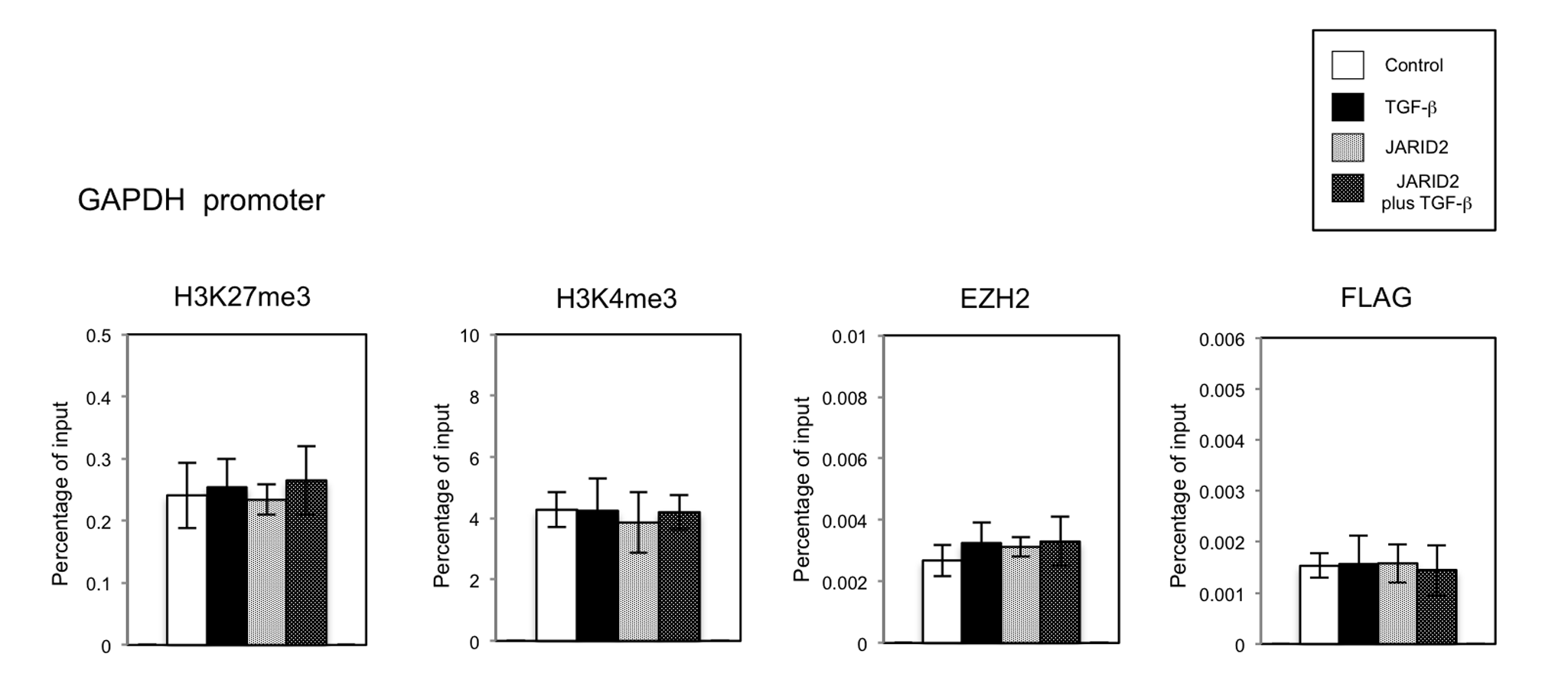


Figure S6. Over-expression of *JARID2* did not affect the histone H3 methylation and EZH2 recruitment on the regulatory region of *GAPDH* gene in A549 cells.

ChIP analyses of H3K27me3, H3K4me3, EZH2 and FLAG-tagged JARID2 on the regulatory region of *GAPDH* gene in A549 cells are shown. The occupancies of methylated histones, EZH2 or FLAG-JARID2 protein on the region were analyzed by quantitative PCR.
